# What clinical crew competencies and qualifications are required for helicopter emergency medical services? A review of the literature

**DOI:** 10.1186/s13049-020-00722-z

**Published:** 2020-04-16

**Authors:** Siobhán Masterson, Conor Deasy, Mark Doyle, David Hennelly, Shane Knox, Jan Sorensen

**Affiliations:** 1 Medical Directorate, National Ambulance Service, Dooradoyle House, Dooradoyle Road, Limerick, V94 HW6E Ireland; 2 Emergency Department, Cork University Hospital, Cork, Ireland; 3 Retired Emergency Medicine Consultant, Waterford, Ireland; 4 National Ambulance Service College, Dublin, Ireland; 5 Healthcare Outcomes Research Centre, Royal College of Surgeons in Ireland, Dublin, Ireland

**Keywords:** Prehospital care, Helicopter retrieval, Clinical assessment, Competence

## Abstract

**Background:**

Patients served by Helicopter Emergency Medical Services (HEMS) tend to be acutely injured or unwell and in need of stabilisation followed by rapid and safe transport. It is therefore hypothesised that a particular clinical crew composition is required to provide appropriate HEMS patient care. A literature review was performed to test this hypothesis.

**Methods:**

MEDLINE, EMBASE, Web of Science and the Cochrane Database of Systematic Reviews were systematically searched from 1 January 2009 to 30 August 2019 to identify peer-reviewed articles of relevance. All HEMS studies that mentioned ‘staffing’, ‘configuration’, ‘competencies’ or ‘qualifications’ in the title or abstract were selected for full-text review.

**Results:**

Four hundred one studies were identified. Thirty-eight studies, including one systematic review and one randomised controlled trial, were included. All remaining studies were of an observational design. The vast majority of studies described clinical crews that were primarily doctor-staffed. Descriptions of non-doctor staff competencies were limited, with the exception of one paramedic-staffed model.

**Conclusions:**

HEMS clinical crews tended to have a wider range of competencies and experience than ground-based crews, and most studies suggested a patient outcome benefit to HEMS provision. The conclusions that can be drawn are limited due to study quality and the possibility that the literature reviewed was weighted towards particular crewing models (i.e. primarily doctor-staffed) and countries. There is a need for trial-based studies that directly compare patient outcomes between different HEMS crews with different competencies and qualifications.

## Background

Helicopter Emergency Medical Services (HEMS) is a component of prehospital emergency care. In common with other Emergency Medical Services (EMS), HEMS is generally required to fulfil one or more of the following objectives:

- To respond to an acutely injured or unwell patient quickly
- To bring emergency medical expertise to an acutely injured or unwell patient
- To transport an acutely injured or unwell patient quickly and safely.

The types of HEMS in operation internationally are generally categorised into doctor-staffed and non-doctor-staffed models. However, the array and experience of clinical staff employed in HEMS means that dichotomising HEMS models into ‘doctor vs. non-doctor’ provides limited information on the qualifications and competencies required to serve HEMS patients. While heterogeneity is a feature of HEMS models, HEMS patients tend to be acutely injured or unwell, and in need of advanced care and/or safe and rapid transport. This suggests that there is a specific/ particular clinical staff model that is required for an effective HEMS model. Therefore, the aim of this study was to perform a review of academic literature to identify the clinical qualifications and competencies HEMS required to provide care that optimises patient outcomes. For the purposes of this review and in the absence of a specific outcome dataset for HEMS treatment, ‘appropriate care’ was determined by the outcomes defined for each study.

## Methods

### Search of literature

A search strategy was devised with the assistance of a research librarian and a search of the literature was conducted from 1 January 2009 to 30 August 2019. The search included all peer-reviewed articles in MEDLINE, including quantitative and qualitative studies, and literature reviews. Conference abstracts/proceedings, grey literature, and articles that were written in a language other than English were excluded. A similar search was performed in both Web of Science and EMBASE. The review question was constructed according to the patients, interventions, comparator and outcome (PICO) strategy recommended by the Preferred Reporting Items for Systematic Reviews and Meta-Analysis Protocols (PRISMA-P) 2015 Checklist [1]:

“In the population of patients who are acutely injured or unwell and attended by a Helicopter Emergency Medical Service, what combination of competencies and qualifications of the clinical crew are required to provide the appropriate level of care?”

Three search terms were built using the ‘Title/Abstract’ and ‘MeSH’ descriptors in MEDLINE; ‘qualifications’; ‘competencies’; and ‘helicopter emergency service’ (see Supplementary file A). A separate search was performed in the Cochrane Database of Systematic Reviews, using only the MeSH descriptor of ‘air ambulances’. All article citations were imported into Endnote Desktop Version X9.2 (Bld 13,018) and duplicate articles were identified and removed using the ‘find duplicates’ function in Endnote. A description of the search strategy is provided in the Supplementary Files.

The review protocol was registered with the international prospective register of systematic reviews PROSPERO (Registration number: CRD42020151104).

### Selection of studies

**Fig. 1:**
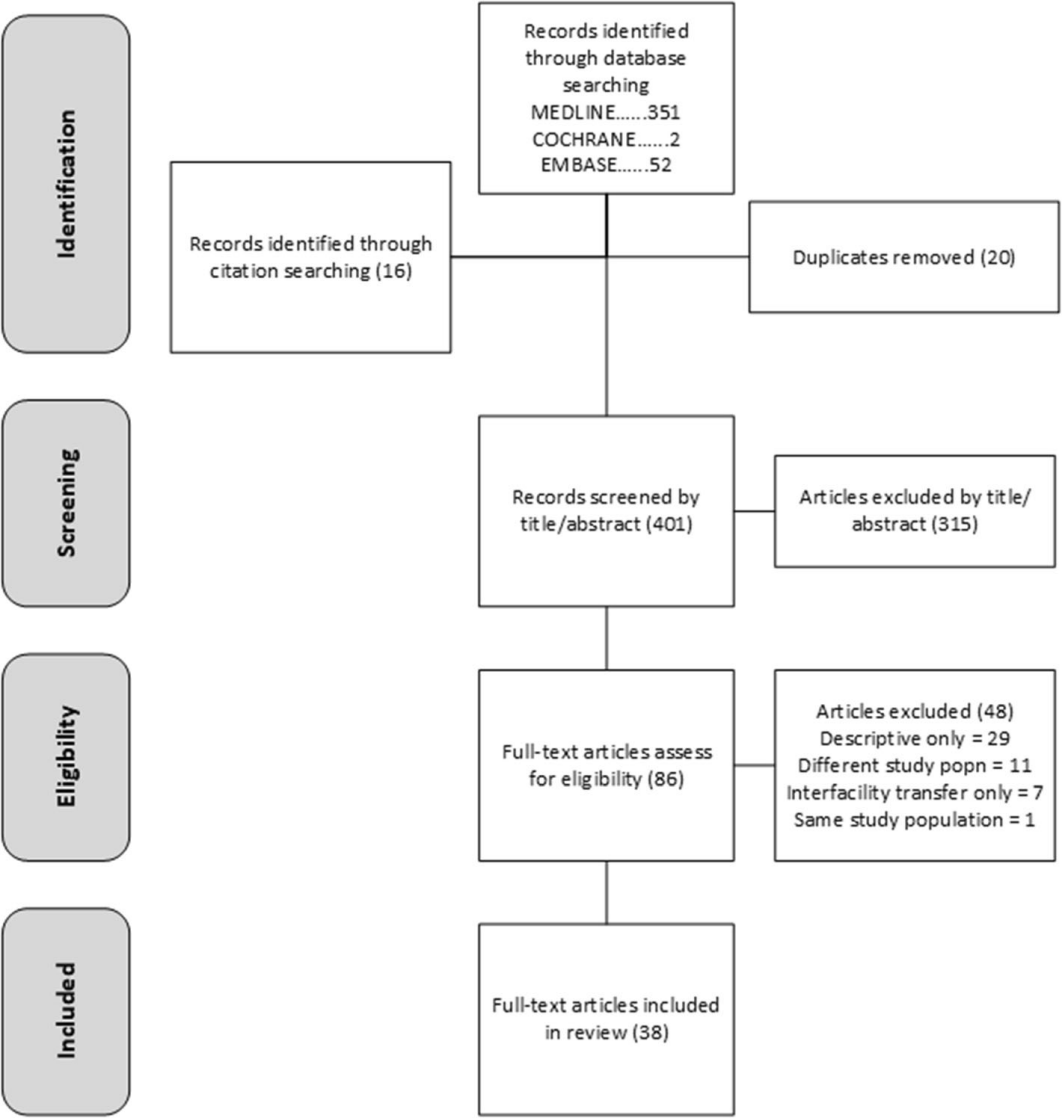
Literature Search Flowchart

Broad search terms were used in order to ensure comprehensive capture of relevant articles. Two reviewers performed the selection of articles based on the review question, initially identifying all articles where the title and/or abstract made specific mention of ‘staffing’, ‘configuration’, ‘competencies’ and/or ‘qualifications’. Case reviews were excluded due to the limited ability to generalise the data from such studies. For studies published using data from the same cohort of patients over the same timeframe and using similar outcome measures, the most recent publication only was included. Instances of disagreement were discussed to meet consensus. Articles that described inter-facility care only were also excluded in order to ensure the primary focus of the review was on emergency care provision. Full-text articles were then downloaded and comprehensively assessed for eligibility (see Fig. 1).

### Data extraction and quality assessment

Full-text articles were reviewed and data was extracted under the following headings:

- Author, date and country
- Study design type
- Clinical crew structure
- Crewing model and staff grade/qualifications
- Patient group served
- Clinical interventions provided
- Outcomes
- Key Results
- Study strengths
- Study weaknesses.

The quality of the randomised controlled trial (RCT), the systematic review and cohort studies, were assessed using critical appraisals checklists as guides (https://casp-uk.net/casp-tools-checklists/). Questionnaire/survey type studies were assessed using the Critical Appraisal of a Cross-Sectional Study (Survey) from the Centre for Evidence-based Management (https://www.cebma.org/wp-content/uploads/Critical-Appraisal-Questions-for-a-Survey.pdf) (see Supplementary Tables 1, 2, 3 and 4).

### Data synthesis

Following a quality review of each full-text article for which data was extracted, data from the remaining articles was tabulated and a narrative synthesis describing commonly identified competencies was produced. This analysis was used to develop conclusions and recommendations about the type of clinical crew needed for HEMS and to identify requirements for further research and analysis. As described in the introduction, because of the degree of heterogeneity in HEMS models internationally, there was also heterogeneity in patient outcomes published. This meant that meta-analysis based on patient outcomes was not performed.

## Results

The search strategy led to the inclusion of 38 articles describing 20 HEMS models from 12 different countries. Five model descriptions included specific mention of a joint HEMS and inter-facility care function: Australia (New South Wales) [2]; Australia (Victoria) [3]; Eastern Denmark [4]; Norway [5]; Sweden (Dalarna) [6]. The remaining 15 models described a HEMS function only.

Of the 38 studies selected for review, there was only one RCT and one systematic review. The remaining studies were observational in nature, including 31 cohort studies and five survey-type descriptive analyses of HEMS activity and clinical crew competencies. With regard to study quality, the RCT was of good quality despite having wide confidence intervals for the treatment effect [7]. However, for the systematic review it was unclear whether the quality of studies included was sufficiently assessed [8]. For the survey-type studies, three were deemed to be of good quality, [5, 9, 10] while two did not report the survey response rate [11, 12]. It was unclear from the final survey whether selection bias may have occurred [12]. Of the 31 cohort studies, study quality varied. Patient recruitment was acceptable and outcome/exposure were accurately measured in all studies. Confounding factors were identified in 16 studies but taken into account in the analysis in only 13 studies. Patient follow-up was incomplete in three studies, [13–15] and insufficiently long in two studies [13, 14]. With regard to result precision, 11 studies only described the population attended, and nine had a small study sample with limited precision or wide 95% confidence intervals for the results.

Five studies presented results with narrow 95% confidence intervals, [16–20] and four provided a sensitivity analysis of result accuracy [15, 21–23]. For most studies, study findings were either novel or fitted with other available evidence.

The systematic review investigated the costs and benefits of HEMS, [8] and the randomised clinical controlled trial described an intervention provided by paramedic/nurse-staffed HEMS [7]. Three studies compared the competencies of HEMS doctors and HEMS paramedics, but did not report on the impact of competencies on patient outcomes [12, 24, 25]. Twelve studies compared patient outcomes between a doctor or paramedic/doctor staffed HEMS and a paramedic/nurse-staffed ground EMS [2, 11, 15, 16, 20–23, 26–29]. Four studies compared intervention and patient outcomes from paramedic-staffed HEMS with paramedic-staffed ground EMS [14, 17, 30, 31]. Six studies compared outcomes from a doctor-staffed HEMS and doctor-staffed ground EMS. One study compared patient outcomes from two different types of HEMS clinical crew configurations [32], and a further study investigated the impact of HEMS doctor involvement in tasking, treatment and transport decisions for paediatric drowning victims [33, 34].

### Variation in HEMS clinical crew qualifications

In jurisdictions where HEMS were either solely or primarily doctor-staffed, there was a difference in medical specialties needed to operate as a HEMS doctor. It should be noted that the specialties of emergency medicine and prehospital emergency medicine are not established in all countries where HEMS models were operational, which may in part explain the emphasis on in-hospital acute care specialties. There was also a vast difference in the competencies of nurses and paramedics employed on HEMS. The range of clinical crew models described in the literature is summarised in Table 1.

**Table 1:** Comparison of HEMS Clinical Crew Staffing Models and Qualifications

Country/HEMS name	Staffing Model	HEMS-Specific Qualifications/Experience
Germany (Eich et al., 2009)	Ambulance–based emergency physician	• Extensive experience in a doctor-staffed ambulance (attended at least 300 incidents as ambulance-based emergency physician) • Hold an Advanced Life Saving Certificate • Completed a 4-month rotation in paediatric anaesthesia
The Netherlands (Gerritse et al., 2010)	Anaesthesiologist or trauma surgeon staffed and specialised nurse	• Board certified trauma surgeon or anaesthesiologist with 6 months’ extra training in adult and paediatric emergency care, pain management and extrication technique • Nurse training not described
Norway (Bjornsen et al., 2018)	Doctor-staffed by anaesthetic flight physicians	• Board certification in anaesthesiology • Experience in paediatric anaesthesiology • Completed a course in trauma care • Have knowledge and proficiency in CPR
Great Western Air Ambulance Service, United Kingdom (Von Vopelius-Feldt, 2014)	Prehospital critical care consultant and critical care paramedic for “80% of shifts”	• Doctors undertake a training programme with “specific competencies and mentored practice, coupled with theoretical and simulation training” • Critical care paramedics “completed a university-based theory and practical training course with mentoring and supervised experience, followed by the successful completion of a comprehensive qualifying assessment.”
Warwickshire and Northamptonshire Air Ambulance, United Kingdom (Fullerton, 2009)	2 crew mixes: doctor and paramedic OR paramedic-paramedic. Dependant on staff availability	• Paramedic crew undergo 40 h’ additional clinical training • Doctors comply with eligibility requirements, including at least registrar level training and extensive training & exposure to acutely ill patients
Bristol Great Western Air Ambulance and Wiltshire Air Ambulance, United Kingdom (Von Vopelius-Feldt, 2014)	2 crew mixes: doctor and paramedic OR paramedic-paramedic. Dependant on staff availability	• Senior registrar or consultant in emergency medicine or anaesthesia • Critical care paramedic with over 5 years’ experience and postgraduate certificate in pre-hospital critical care
Midlands Air Ambulance, United Kingdom (McQueen, 2015)	• Doctor-staffed for high severity trauma • Paramedic-staffed for support of ambulance crews when doctor unavailable or call would not benefit from doctor intervention	• Paramedics “have received additional training and operate as critical care paramedics.” • Doctor is senior trainee in emergency medicine, critical care or anaesthesia and has undergone specialist training to deliver enhanced prehospital care, RSI
Suwon, South Korea (Jung, 2016)	• Multi-disciplinary staff for severe trauma (5 trauma surgeons, 1 emergency physician, a nurse practitioner and emergency technician • Emergency technician staffed for minor injuries in inaccessible locations	• Emergency technicians give basic life support procedures with phone support from the hospital medical team
Japan (Abe, 2014)	• Doctor and nurse staffed	• No specific details provided
Air Ambulance Victoria, Australia (Andrew, 2015)	• Intensive Care Flight Paramedic and air crewman	• Existing Intensive Care Paramedics complete an additional 9-months’ postgraduate training in aeromedical rescue. Also acquire skills including paediatric RSI, mechanical ventilation, insertion of arterial lines and invasive monitoring, administration of a wider range of medications • Air crewmen have 120 h training to fulfil the role of Emergency Medical Technician
Greater Sydney Area HEMS, Australia (Burns, 2017)	• Doctor and paramedic staffed	• Doctors are board-certified senior registrars from Emergency Medicine or Anaesthesia; minimum of 5 years’ postgraduate experience • Paramedics are critical care specialists with a minimum of 10 years’ experience and additional training in pre-hospital and retrieval medicine.
East Denmark (Afzali, 2013)	• Doctor and paramedic staffed	• Consultant anaesthesiologist experienced in intensive care pre-hospital • Paramedic with special training in navigation and radio communication techniques.
Central Denmark (Rognås, 2013)	• Doctor and EMT staffed	• Anaesthesiologists with at least 4.5 years’ experience in anaesthesia. All work in and outside operating theatre as part of their daily work.
Finland (Heinanen, 2018)	• Doctor staffed	• Mainly anaesthesiologists specialised in emergency care
France (Desmettre, 2012)	• Team from hospital led by emergency physician	• No details provided
Dalarna, Sweden (Kornhall, 2018)	• Doctor and HEMS crewmember	• Doctor has board certification in anaesthesiology • HEMS crewmember is registered pre-hospital nurse
Pittsburgh, United States (Sperry, 2018)	• Paramedic and flight nurse staffed	• Not described

### Clinical competencies added by different clinical crew models

Andrew and colleagues described advanced procedures carried out by Intensive Care Flight Paramedics (ICFPs) in Victoria and highlighted the successful application of pain reduction, rapid sequence induction (RSI) and administration of red blood cell products [3]. Von Volpelius-Feldt et al. identified a range of additional competencies brought by critical care paramedics and HEMS doctors, but highlighted the value of doctor-CCP team working in facilitating critical care paramedic practice [12]. This theme of combined competencies was continued by Van der Eng and colleagues, who used a Delphi process to devise three indicators of quality in the management of patients with poly-trauma, i.e. education, exposure and experience [15]. In a sample of 442 patients, they estimated that 220 patients were treated by a fully competent team i.e. fulfilled the three indicators of competency. Van Schuppen and Bierens initially identified the additional skills that a doctor added to HEMS, [29] and in a later study identified that doctors added qualitative skills that were less tangible such as clinical judgement and impact on decision-making [11]. However, while the studies described enhanced competencies provided by different types of HEMS clinical crews, they did not estimate the impact of these additional competencies on patient outcomes. Competencies identified in the literature are presented in Table 2.

**Table 2:** Individual Competencies identified categorised by Country or Jurisdiction

Country and References	Competencies	
UK (Fullerton et al., 2009, Shapey et al., 2012, McQueen et al., 2013, McQueen et al., 2015a, von Vopelius-Feldt and Benger, 2014, Smith et al., 2019)	• ACLS • Amputation (no instance of practice recorded) • Chest drain • Cricothyroidotomy • Epi admin • ETI in cardiac arrest • External jugular access • External pacing • Fascia iliaca block • IO access • IV Etomidate • IV Ketamine administration • IV Propofol • IV Suxamethonium	• Management of paralysed patient • Mag sulphate in cardiac arrest • Needle chest decompression • Peri-mortem Caesarean section • Procedural sedation • Fluid resuscitation • Rocuronium intravenous • RSI • Surgical airway • Thoracostomy • Thoracotomy • Torsades de pointes arrythmia • Venous cut-down • Wave form capnography • Large joint reduction
Victoria, Australia (Heschl et al., 2018b, Andrew et al., 2015, Heschl et al., 2018, Meadley et al., 2016)	• Advanced analgesia • Blood-gas analysis • Blood transfusion • Comprehensive analgesia options including opiods and ketamine • Cricothyroidotomy	• Paediatric RSI with suspected TBI • RSI – adult and paediatric • Thoracostomy • Transfusion of Red Cell Concentrates • Vasoactive medication admin • IO access
United States(Sperry et al., 2018, Kashyap et al., 2016, Polites et al., 2017)	• Airway management • ATLS • IV fentanyl and morphine administration • IV fluid administration	• Inter-hospital transfer of unstable medical patients Plasma transfusion • Spinal immobilisation • Ventilation • Transportation of severe trauma patients
Germany (Eich et al., 2009)	• Analgesia/Sedation • Catecholamine administration • Chest tube and drain– paediatric and adult • CPR	• Defibrillation– paediatric and adult • IO access– paediatric and adult • Intubation – paediatric and adult • Volume administration
Denmark (Rognås et al., 2013)	• Drug-assisted airway management (non RSI) • RSI intubation	• Nasopharyngeal airway • Surgical airway
New South Wales(Burns et al., 2017, Garner et al., 2016)	• Analgesia/procedural sedation • Direct screening of emergency calls to identify appropriate (paediatric) response • Regional anaesthesia/nerve block • RSI and intubation – adult and paediatric • Surgical airway	Adult EZ-intraosseous access • Blood transfusion • Orthopaedic manipulation of joint/limb • Use of ultrasound (diagnostic/procedural) • Hypertonic saline administration • Thoracostomy/chest drain
Norway(Bjornsen et al., 2018, Johnsen et al., 2017)	• ACLS • Anti-arrythmic therapy • Arterial line insertion • BMV adult/paediatric • Chest tube placement and drainage • Central venous catheter insertion • Dislocated joint reposition • ETI adult/paediatric • Fracture reposition	• Gastric tube insertion • Incubator transport • Inhalation therapy • Invasive and non-invasive ventilation • IV/IO access • Major incident management • Reduction and immobilisation of fractures • RSI • Umbilical cord catheterisation

Country	Competencies		
*The Netherlands (van Schuppen and Bierens, 2015, van Schuppen and Bierens, 2011, Ketelaars et al., 2018, Gerritse et al., 2010, Franschman et al., 2012)	• Analgesia/Sedation • Catecholamine administration • Chest tube • CPR • Drug-assisted and non-drug-assisted ETI • Echocardiography • Extrication techniques • Intubation • RSI intubation • Volume administration Diagnostic competencies • Cold diuresis • Diaphragm rupture • Hypocalcaemia • Hypomagnesemia • Kidney failure • Malignant hyperthermia • Tracheobronchial injury Therapeutic competencies • Amputation • Atracurium • Blood transfusion • Caesarean section • Calciumchlorid Cefuroxime • Chest tube • Cricothyrotomy (surgical • Dopamine • Ephedrine • Escharotomy • Etomidate	• Fascia iliaca compartment block • Flumazenil • Gum elastic bougie • Hydrocortisone • Hydroxycobalamine • HyperHaes® • Incision • Insulin • Intravenous access, central • Jet ventilation • Lidocaine • Laryngeal Mask Airway (LMA®) • Magnesium • Mannitol • Nasopharyngeal airway • Noradrenaline • Pericardiocentesis • Potassium • Procainamide Propofol • Push foreign object from trachea into bronchus • Rocuronium • Ropivacaine • Succinylcholine • Sufentanil • Supraglottic airway • Suturing • Thoracotomy • Tracheotomy • Trachlight • Thrombolysis • Venesection	Clinical judgment competencies • Advance endotracheal tube in case of bronchus rupture • Cardiopulmonary bypass in hypothermia • Dialysis in hyperkalemia • Induction with s-ketamine in asthma/COPD • Intravenous lidocaine administration before endotracheal intubation in possible intracranial hypertension • Intubation and ventilation in pneumonia • Magnesium in bronchial asthma/COPD • Push foreign object in further in bronchus • Resuscitation in hypothermia is beneficial • Supraglottic airway in “cannot intubate, cannot ventilate” situation • Thrombolysis in pulmonary embolus

*full list of competencies identified in Van Schuppen et al *(2011) available at**http://links.lww.com/EJEM/A2**and**http://links.lww.com/EJEM/A3*

### Impact of HEMS clinical crew qualifications and competencies on patient outcomes and successful interventions

As stated above, studies comparing HEMS to ground-based EMS suggested better patient outcomes, although it should be remembered that the studies reviewed had an observational design. In studies where the HEMS and ground-based EMS had similar qualifications, for patients suffering severe trauma, HEMS intervention was almost invariably associated with better patient outcomes. For example, in Germany, where both HEMS and ground-based EMS are doctor-staffed and have similar clinical competencies, two studies showed that HEMS was associated with an adjusted decrease in in-hospital mortality [18, 20]. In a French study, where doctor-staffed mobile intensive care units (MICUs) were deployed to patients with severe blunt trauma either by road or helicopter, adjusted mortality was lower for patients attended by helicopter-based MICU compared with those attended by a road-based unit [19]. In two US studies using data from the National Trauma bank, paramedic/nurse-staffed HEMS was associated with improved patient outcomes when compared to paramedic-staffed ground-based EMS [17, 31]. Similarly, a further US study suggested that while HEMS patients tended to be sicker, HEMS provision enabled better adherence to sepsis guidelines due to shorter transport times [30].

In HEMS models where the qualifications of the HEMS crew were more advanced than those of the ground-based EMS crew, studies also suggested that HEMS improved patient outcomes. In New South Wales where HEMS was doctor-staffed, it was estimated that the adjusted odds of dying in hospital was three to four times higher for ground-transported adult patients who suffered major trauma when compared to HEMS-treated patients [23]. Also in New South Wales, when the identification and triaging of paediatric drowning patients by HEMS doctors was discontinued, this resulted in incorrect transport of paediatric patients to adult facilities [33]. While statistical significance was not reached due to small numbers, prehospital rapid sequence induction (RSI) intubation by ICFPs in Victoria was associated with a shorter hospital stay and more favourable six-month functional outcome, when compared with usual care by ground crews who did not perform intubation [14]. In a propensity-matched analysis of data from the Japan Trauma Bank, doctor-staffed HEMS were associated with improved odds of survival, [16] as was also the case in South Korea [32]. In the Netherlands, where advanced medical procedures are restricted to doctor-staffed HEMS, Ringburg and colleagues estimated that HEMS was both cost-effective and responsible for saving an additional 29 lives following severe trauma over a four-year period (2003–2006) [22]. A later Dutch study in a single centre confirmed these findings [21]. Finally, the PHANTOM study in the UK concluded that HEMS that were staffed by an enhanced care team (ECT) of doctors and critical care paramedics (CCPs) had a statistically significant benefit in adjusted survival rates for severe trauma patients when compared to patients solely treated by a ground-based paramedic [28].

## Discussion

This literature review suggests that HEMS clinical crews have a wide array of competencies and experience. However, even in scenarios where the HEMS crew qualifications and competencies are similar to the ground crew, the studies included suggest an additional advantage to HEMS-provided care for patients. Due to the heterogeneity of study types and differences in ground crew competencies in different jurisdictions, and the limited patient outcomes reported in the literature, it is unclear what type of clinical crew model is best suited for HEMS provision. It is of note that previous research has focused on specific interventions (e.g. intubation success) and that this type of intervention-specific study has provided evidence for one type of crew model compared to another [35, 36]. While most of the studies in this review suggested a benefit to HEMS provision – regardless of the competencies of ground EMS crews – it is important to note that the majority of studies included were observational in nature, and no randomised controlled trials comparing one type of HEMS clinical crew model with another were identified. There continues to be a need for a ‘conceptual framework’ to guide researchers in estimating the benefit of different HEMS crews with different qualifications and competencies [8].

### Limitations

The literature identified in this review contained very limited data comparing different crew qualifications and competencies. This means that while descriptions of the clinical crew composition and competencies were provided, the association between the clinical crew type and patient outcome is unclear. Additionally, there was probable publication bias towards doctor-staffed HEMS models as evidenced by the low number of US and Canadian models represented in the search results. Studies included were primarily of low grade, with only two studies describing trial results. Additionally, while the review provides descriptions of practice in Australia and Europe, there appears to be limited availability of published academic literature from the United States, Canada, and other international jurisdictions. In summary, due to the fact that literature on this subject is limited in availability and poorly indexed, it is exceedingly difficult to come to definitive conclusions about the type of clinical crew qualifications and competencies will best serve HEMS patients.

## Conclusion

The majority of studies included in this literature review suggest that HEMS confers a patient benefit, regardless of whether the clinical crew composition is similar or more advanced than the ground-based EMS clinical crew composition. However, the quality of evidence identified highlights the need for trial-based study designs that directly compare patient outcomes following different HEMS crews to be established. There is also a need to ensure that the evidence base is representative of international HEMS models and not weighted towards particular countries or clinical crew models (i.e. doctor-led). It is acknowledged that establishing this type of quality evidence base will be challenging, but pragmatic ways to address this research question could be pursued through collaboration between HEMS providers internationally. The provision of HEMS has become a common component of overall EMS service provision. Establishing the HEMS clinical crew competencies and qualifications that is of most benefit to acutely unwell or injured patients is an important next step in the appropriate development of this emergency service.

## Supplementary information

**Additional file 1: Table S1.** Quality Assessment of Randomised Controlled Trial **Table S2.** Quality Assessment of Systematic Review **Table S3.** Quality Assessment of Survey-Type Studies **Table S4.** Quality Assessment of Cohort Studies

## Data Availability

Not applicable.
