# Supplementary material for: What clinical crew competencies and qualifications are required for helicopter emergency medical services? A review of the literature
Source: Scand J Trauma Resusc Emerg Med. 2020 Apr 16;28:28. doi: 10.1186/s13049-020-00722-z (PMC7164232; doi:10.1186/s13049-020-00722-z)
Supplement: Supplementary file 1 — Additional file 1: Table S1. Quality Assessment of Randomised Controlled Trial Table S2. Quality Assessment of Systematic Review Table S3. Quality Assessment of Survey-Type Studies Table S4. Quality Assessment of Cohort Studies [file 13049_2020_722_MOESM1_ESM.docx]

**Supplementary Tables**

**Table 1: Quality Assessment of Randomised Controlled Trial**

|  | Sperry et al |
| --- | --- |
| Did the trial address a clearly focused issue? | YES |
| Was the assignment of patients to treatments randomised? | YES |
| Were all of the patients who entered the trial properly accounted for at its conclusion? | YES |
| Were patients, health workers and study personnel ‘blind’ to treatment? | Not possible to blind health workers to intervention. Treatment assignments were concealed from data analysts |
| Were the groups similar at the start of the trial | Not completely |
| Aside from the experimental intervention, were the groups treated equally? | YES |
| How large was the treatment effect? | Mortality at 30 days was significantly lower in the treatment group (23.2% vs. 33.0%; difference -9.8%, 95%CI [-18.6% TI -1.0%]) |
| How precise was the estimate of the treatment effect? | Wide 95% CIs |
| Can the results be applied to the local population, or in your context? | Possibly, if paramedic/nurse-staffed HEMS is provided with medical oversight |
| Were all clinically important outcomes considered? | YES |
| Are the benefits worth the harms and costs? | Decreased mortality suggest intervention of benefit |

**Table 2: Quality Assessment of Systematic Review**

|  | Taylor et al |
| --- | --- |
| Did the review address a clearly focussed question? | YES |
| Did the authors look at the right type of papers? | YES |
| Do you think all the important, relevant studies were included? | YES |
| Did the review's authors do enough to assess the quality of the included studies? | UNCLEAR |
| If the result of the review have been combined, was it reasonable to do so? | YES |
| What are the overall results of the review? | While some studies found HEMS to be cost effective, heterogeneity in studies and systems meant it was difficult to generalise results |
| How precise are the results? | No measure of precision or sensitivity described |
| Can the results be applied to the local population? | Difficult due to degree of heterogeneity in studies and systems |
| Were all important outcomes considered? | YES |
| Are the benefits worth the harms and costs? | Lack of strong evidence to support HEMS but better quality data needed to assess cost-benefit |

**Table 3: Quality Assessment of Survey-Type Studies**

|  | Bjornsen et al | Johnsen et al | Littlewood et al | van Schuppen et al | von Vopelius-Feldt et al |
| --- | --- | --- | --- | --- | --- |
| Did the study address a clearly focused question / issue? | YES | YES | YES | YES | YES |
| Is the research method (study design) appropriate for answering the research question? | YES | YES, consensus process used to identify appropriate questions | YES | YES | YES |
| Is the method of selection of the subjects (employees, teams, divisions, organizations) clearly described? | YES | YES | YES | YES | YES |
| Could the way the sample was obtained introduce (selection)bias? | NO | NO | NO | NO | UNCLEAR |
| Was the sample of subjects representative with regard to the population to which the findings will be referred? | YES | YES | YES | YES | UNCLEAR |
| Was the sample size based on pre-study considerations of statistical power? | Population-based survey | Population-based survey | 100% - sample | NO | NO |
| Was a satisfactory response rate achieved? | 76% - satisfactory | 70% - satisfactory | 100% - satisfactory | Not described | Not described |
| Are the measurements (questionnaires) likely to be valid and reliable? | YES | YES | YES | YES | YES |
| Was the statistical significance assessed? | NO | NO | NO | Not applicable | NO |
| Are confidence intervals given for the main results? | NO | NO | NO | Not applicable | NO |
| Could there be confounding factors that haven’t been accounted for? | YES | NO | NO | NO | YES |

**Table 4: Quality Assessment of Cohort Studies**
